# A publishing pandemic during the COVID-19 pandemic: how challenging can it become?

**DOI:** 10.3325/cmj.2020.61.79

**Published:** 2020-04

**Authors:** Lea Škorić, Anton Glasnović, Jelka Petrak

**Affiliations:** 1 University of Zagreb School of Medicine, Zagreb, Croatia *lea.skoric@mef.hr*; 2 *Croatian Medical Journal*, Zagreb, Croatia

The strategies for managing the health crisis triggered by the COVID-19 pandemic differ from country to country, but “*there has never been such a rapid global collective effort to fight one disease*” (1). The scientific community responded to the crisis by extensive mobilization of all research resources with an aim to shed light on the virus characteristics, mechanisms of its transmission, clinical aspects of the disease, and prevention and management strategies. The number of articles arising from clinical studies and observations has so far multiplied at an unprecedented rate. The largest number has been published in scientific journals, which have responded to this epidemiological and information crisis in accordance with their role in the transmission of new scientific information – quickly, openly, effectively, and responsibly (2).

In the past three months, the medical information system has been hit by a storm marked by a large daily increase in published articles, accelerated review processes, curated hubs for easy and effective access, greater attention to preprint platforms, and free access to all pandemic-related articles.

A good example of a tool for keeping up with the published SARS-CoV-2/COVID-19 articles is LitCovid – a curated literature hub for tracking up-to-date scientific information about the 2019 novel coronavirus (LitCovid: *https://www.ncbi.nlm.nih.gov/research/coronavirus*/). By April 21st, it provided central access to 5761 relevant articles in PubMed, and the number is growing daily. The articles are further categorized by research topics and geographic locations for improved access (3). The growth curve of the number of new publications being included in the database begins to resemble the contagion curve (Figure 1)

In the week of April 13-19, the database included a whopping 1608 articles. Additional five hundred non peer-reviewed articles were posted on several of the most important preprint services. Most of the articles included in LitCovid were published by traditionally leading medical journals – *The* *British Medical Journal* (BMJ), *The Lancet*, *Nature*, *the* *New England Journal of Medicine*, *JAMA*, etc. These articles are mostly from the fields of general information and prevention, control, and management strategies, while in the fields of transmission mechanism, diagnosis and treatment, the leading journal is *Journal of Medical Virology.* One of the journals with a large number of published articles is *Zhonghua Liu Xing Bing Xue Za Zhi* (*Chinese Journal of Epidemiology*).

Why do most influential journals publish the most of these articles? Is it because the authors, knowing that the issue is new, aspire to the best journals, reckoning that their works will be more noticed and they will add another “badge” to their CVs? Or do these reputable journals, by advancing knowledge on COVID-19, want to reinforce their role in the information aspect of this crisis? Or do reputable journals simply have the infrastructure (editors, reviewers, funds) that can handle this pressure? Probably the answer lies in the combination of these factors. However, the question is whether these journals lowered their criteria and whether the “mitigated” review process was a reason for accepting articles that would not, under “normal” circumstances, be accepted in the journals known for their high manuscript rejection rate. And what will be decisive in stopping this publishing inflation? Scientific scrutiny, market, or public interest?

The scientific community has started to exercise caution regarding the relationship between the benefits of the rapid transfer of new insights and the potential harm from the diminished quality of published articles due to the expedited review process and methodological and reporting shortcomings (4). The time between the initial submission and final acceptance is much lower than usually reported (5), while the research is often superficial, poorly designed, and underpowered (6). According to the Retraction Watch Database, some of these have already been retracted.

Before the outbreak of this epidemic, preprint servers with medical articles were not as important as in some other scientific disciplines, such as physics. The reason can be found in the reluctance of the clinical medical community to publish unverified medical information that can bring harm if doctors change clinical guidelines or patients try treatments on their own based on findings that have not been vetted by peer reviewers (7). In recent months, this has changed, and the medRxiv, bioRxiv, SSRN's eLibrary, and Research Square platforms publish several hundred articles daily, accelerating the communication of new results. medRxiv points out that preprints are preliminary reports that have not been peer reviewed and that all manuscripts undergo a basic screening process for offensive or non-scientific content and for material that may pose a health risk, while simultaneously being checked for plagiarism. Nevertheless, these articles should not be relied on to guide clinical practice or health-related behaviors and should not be reported in news media as established information. The latter happens, however, on a daily basis, and the scientific community warns about the potential consequences of publishing such information (6).

Even prestigious journals (eg, *Lancet*, the *BMJ*) now allow the sharing of important findings before peer review, and the use of preprint platforms does not jeopardize the future of peer-reviewed publication (8).

Although Ingelfinger noted in 1977 that “*medicine has become the stuff of headlines*” (9), the results of scientific research have never had such a media impact as today. On the one hand, this has positive effects. The media spotlight has turned to medical professionals, who are reporting on a daily basis on current epidemic developments. Epidemiologists and infectious diseases specialists have become the new type of influencers, and the public partly bases their views on the information thus obtained. On the other hand, social networks and other media may also misinform, so caution is being exercised in the scientific community toward sources and interpretations of scientific facts in the media space.

Publication in open access serves the public good, and information on SARS-CoV-2 and COVID-19 is available free of charge to anyone in the epidemic research and control system. Now, we can finally experience what it would be like to live in a world based on free-for-all medical information. It began with the initiative of Free Read, which in early February launched the petition Unlock Coronavirus Research for the world's scientists, to which reputable medical publishers (Elsevier, Taylor & Francis, Wiley, Springer and others) soon responded. The petition stated that “unlocking” articles published in commercial journals and widening access to all research data are a moral imperative of the coronavirus crisis. Publishers soon created separate portals or dedicated sites with open access to the consolidated articles of their journal portfolio. Academic social networks responded similarly, creating COVID-19 user communities.

Rapid collection and sharing of available research data are enhanced by freely available datasets. One of the largest is the COVID-19 Open Research Dataset (CORD-19). The European Commission with several partners (University of Zagreb Computing Center being one of them) has just launched a European COVID-19 Data Platform as well.

According to many indicators, Croatia has managed this public health crisis successfully. The results of various major or minor studies of epidemiological and clinical nature will certainly emerge in the near future. For now, PubMed shows 18 articles on some of the aspects of COVID-19 published by Croatian authors alone or in collaboration with foreign authors (Supplementary Table 1(Supplementary Table 1)). These are mostly position statements and opinion articles. More than half (10/18) were published in high-impact journals, consistent with our findings that articles related to the COVID-19 pandemic are largely published by the most influential medical journals.

*Croatian Medical Journal* (*CMJ*) responded immediately to the pandemic by an editorial in the February issue requiring urgent epidemiologic surveillance (10). Analyses on the COVID-19 crisis management are yet to be published, and any manuscript on the subject is welcome. The open access policy advocated and implemented by the *CMJ* for more than 15 years (11) will ensure that the published works have permanent, unrestricted, and free access. In addition, since our goal is not to compete with the largest and most influential journals, our publications will continue to be carefully peer reviewed. By this we do not claim that our work is of better or lesser quality, but only specific to the level we aspire to and the audience we address.

The large amount of new scientific information, or “sci-infodemic,” mostly published in medical journals brought about by this global health crisis, and its impact on the professional community and the general public, have made scientific research and its applications one of the main focuses of public policies. They have also shown, in an unprecedented way, all the benefits of open access in the transfer and reception of new scientific knowledge. At this point, it should be emphasized that well designed systematic reviews are particularly important to the professional medical audience in coping with this explosion of information. However, some authors warn that the methodological quality of most of the systematic reviews on previous coronavirus outbreaks is still unsatisfactory, and that those on COVID-19 have higher risk of poor quality, possibly because of the rapid actions performed to conduct them (12). The importance of information literacy and critical reception of published information in this crisis should also be emphasized.

Can the positive changes observed in the way medical journals cope with the information crisis caused by COVID-19 be sustained once we emerge from the crisis? Can the review process be expedited? Can publishers allow more articles on preprint servers and more community-based peer reviews? Can journal editorial boards continue this new practice by publishing OA (free of charge) articles that provide key information for public health or on the most common diseases? We will not have to wait long for the answers.

**Table:** Figure 1. The number of publications added to LitCovid per week.
