# Supplementary material for: A publishing pandemic during the COVID-19 pandemic: how challenging can it become?
Source: Croat Med J. 2020 Apr;61(2):79–81. doi: 10.3325/cmj.2020.61.79 (PMC7230425; doi:10.3325/cmj.2020.61.79)
Supplement: Supplementary Table 1 [file CroatMedJ_61_s008.pdf]

Supplementary Table 1

Papers on SARS-CoV-2/Covid-19 indexed in PubMed with authors affiliated to Croatia (on April 21st)	JCR rank
Ljubicic N, Stojsavljevic-Shapeski S, Virovic-Jukic L, Nikolic M.<br>Plexiglass barrier box to improve ERCP safety during the COVID-19 pandemic<br>Gastrointest Endosc. 2020 Apr 16:S0016-5107(20)34172-9. doi: 10.1016/j.gie.2020.04.020.	Q1
Barazzoni R, Bischoff SC, Breda J, Wickramasinghe K, Krznaric Z, Nitzan D, Pirlich M, Singer P.<br>ESPEN expert statements and practical guidance for nutritional management of individuals with SARS-CoV-2 infection<br>Clin Nutr. 2020 Mar 31:S0261-5614(20)30140-0. doi: 10.1016/j.clnu.2020.03.022.	Q1
Gralnek IM, Hassan C, Beilenhoff U, Antonelli G, Ebigbo A, Pellisè, Arvanitakis M, Bhandari P, Bisschops R, Van Hooft JE, Kaminski MF, Triantafyllou K, Webster G, Pohl H, Dunkley I, Fehrke B, Gazic M, Gjergek T, Maasen S, Waagenes W, de Pater M, Ponchon T, Siersema PD, Messmann H, Dinis-Ribeiro M.<br>ESGE and ESGENA Position Statement on gastrointestinal endoscopy and the COVID-19 pandemic<br>Endoscopy. 2020 Apr 17. doi: 10.1055/a-1155-6229.	Q1
Ćosić K, Popović S, Šarlija M, Kesedžić I.<br>Impact of Human Disasters and COVID-19 Pandemic on Mental Health: Potential of Digital Psychiatry<br>Psychiatr Danub. 2020 Spring	Q4
Mindoljević Drakulić A, Radman V.<br>Crisis Psychodrama in the Era of COVID-19<br>Psychiatr Danub. 2020 Spring	Q4
Marčinko D, Jakovljević M, Jakšić N, Bjedov S, Mindoljević Drakulić A.<br>The Importance of Psychodynamic Approach during COVID-19 Pandemic<br>Psychiatr Danub. 2020 Spring	Q4
Jakovljevic M, Bjedov S, Jaksic N, Jakovljevic I.<br>COVID-19 Pandemia and Public and Global Mental Health from the Perspective of Global Health Security<br>Psychiatr Danub. 2020 Spring	Q4
Jakovljevic M.<br>COVID-19 Crisis as a Collective Hero's Journey to Better Public and Global Mental Health<br>Psychiatr Danub. 2020 Spring	Q4

Goren A, Vano-Galvan S, Wambier CG, McCoy J, Gomez-Zubiaur A, Moreno-Arrones OM, Shapiro J, Sinclair R, Gold MH, Kovacevic M, Mesinkovska NA, Goldust M, Washeni K.<br>A preliminary observation: male pattern hair loss among hospitalized COVID-19 patients in Spain - A potential clue to the role of androgens in COVID-19 severity<br>J Cosmet Dermatol. 2020 Apr 16. doi: 10.1111/jocd.13443.	Q4
Jakovac H.<br>COVID-19 and vitamin D-Is there a link and an opportunity for intervention?<br>Am J Physiol Endocrinol Metab. 2020 May 1	Q1
Lippi A, Domingues R, Setz C, Outeiro TF, Krisko A.<br>SARS-CoV-2: at the crossroad between aging and neurodegeneration<br>Mov Disord. 2020 Apr 15. doi: 10.1002/mds.28084.	Q1
Wolkewitz M, Puljak L.<br>Methodological challenges of analysing COVID-19 data during the pandemic<br>BMC Med Res Methodol. 2020 Apr 14	Q2
Emanueli C, Badimon L, Martelli F, Potočnjak I, Carpusca I, Robinson EL, Devaux Y.<br>Call to action for the cardiovascular side of COVID-19<br>Eur Heart J. 2020 Apr 13:ehaa301. doi: 10.1093/eurheartj/ehaa301.	Q1
Lauc G, Sinclair D.<br>Biomarkers of biological age as predictors of COVID-19 disease severity<br>Aging (Albany NY). 2020 Apr 8. doi: 10.18632/aging.103052.	Q1
Borges do Nascimento IJ, Cacic N, Abdulazeem HM, von Groote TC, Jayarajah U, Weerasekara I, Esfahani MA, Civile VT, Marusic A, Jeroncic A, Carvas Junior N, Pericic TP, Zakarija-Grkovic I, Meirelles Guimarães SM, Luigi Bragazzi N, Bjorklund M, Sofi-Mahmudi A, Altujjar M, Tian M, Arcani DMC, O'Mathúna DP, Marcolino MS.<br>Novel Coronavirus Infection (COVID-19) in Humans: A Scoping Review and Meta-Analysis<br>J Clin Med. 2020 Mar 30	Q1
Jakovac H.<br>COVID-19 - is the ACE2 just a foe?<br>Am J Physiol Lung Cell Mol Physiol. 2020 Apr 1. doi: 10.1152/ajplung.00119.2020.	Q1
Lippi G, Simundic AM, Plebani M.<br>Potential preanalytical and analytical vulnerabilities in the laboratory diagnosis of coronavirus disease 2019 (COVID-19)	

Clin Chem Lab Med. 2020 Mar 16:/j/cclm.ahead-of-print/cclm-2020-0285/cclm-2020-0285.xml. doi: 10.1515/cclm-2020-0285.	Q1
Čivljak R, Markotić A, Kuzman I.<br>The third coronavirus epidemic in the third millennium: what's next?<br>Croat Med J. 2020 Feb 29	Q2
